# Fluorescence Switchable Conjugated Polymer Microdisk Arrays by Cosolvent Vapor Annealing

**DOI:** 10.3390/polym13020269

**Published:** 2021-01-15

**Authors:** Hiroshi Yamagishi, Tokiya Matsui, Yusuke Kitayama, Yusuke Aikyo, Liang Tong, Junpei Kuwabara, Takaki Kanbara, Masakazu Morimoto, Masahiro Irie, Yohei Yamamoto

**Affiliations:** 1 Department of Materials Science, Faculty of Pure and Applied Sciences, University of Tsukuba, 1-1-1 Tennodai, Tsukuba 305-8573, Ibaraki, Japan; yamagishi.hiroshi.ff@u.tsukuba.ac.jp (H.Y.); s1711061@s.tsukuba.ac.jp (T.M.); s-kitayama@ims.tsukuba.ac.jp (Y.K.); aikyoy@sc.sumitomo-chem.co.jp (Y.A.); tong.l.aa@m.titech.ac.jp (L.T.); kuwabara@ims.tsukuba.ac.jp (J.K.); kanbara@ims.tsukuba.ac.jp (T.K.); 2 Tsukuba Research Center for Energy Materials Science (TREMS), Faculty of Pure and Applied Sciences, University of Tsukuba, 1-1-1 Tennodai, Tsukuba 305-8573, Ibaraki, Japan; 3 Department of Chemistry and Research Center for Smart Molecules, Rikkyo University, Nishi-Ikebukuro 3-34-1, Toshima-ku, Tokyo 171-8501, Japan; m-morimoto@rikkyo.ac.jp (M.M.); iriem@rikkyo.ac.jp (M.I.)

**Keywords:** microdisk, self-assembly, array, fluorescence, switching

## Abstract

Depositing minute light emitters into a regular array is a basic but essential technique in display technology. However, conventional lithographic methodologies involve multistep and energy-consuming processes. Here, we develop a facile method in which organic and polymeric fluorescent dyes spontaneously aggregate to form a patterned microarray. We find that a thin film of fluorescent π-conjugated polymer transforms into micrometer-sized aggregates when exposed to binary organic vapor at ambient temperature. The arrayed microaggregates can be formed over the whole substrate surface when using a quartz substrate that is prepatterned with regular hydrophilic boxes and hydrophobic grids. The resultant microarray is applicable to optical memories and displays when photoswitchable fluorophores are doped into the polymer matrix.

## 1. Introduction

Regular arrays of micro- and nanometer-sized devices integrated with light emitters enable the multipixeled displaying or collective assay of trace amounts of chemical or biological compounds [1,2,3]. The structural and electrical complexity of these arrays are usually realized by means of photo- or electron-beam lithography techniques. However, these top-down techniques are costly in terms of, for instance, the facilities required for the fabrication, the time and labor devoted for multistep processing, and the chemicals utilized for the protecting and rinsing steps. These drawbacks can be readily circumvented by using their counterpart bottom-up technology. The bottom-up method relies on the self-assembling behavior of small molecules and thus requires a low energy and simple procedure, which is especially useful for the fabrication of submicron-scale objects. In this field, a major focus is currently put on the arbitral molding and positioning of the nano-objects over a wide area [4,5,6]. Several successful examples have recently been reported, but they typically involve complex devices and enormous energy and time consumption during the fabrication process [7,8].

In this article, we report a facile method for the fabrication of regular device arrays by combining the advantages of the top-down and bottom-up strategies. We arbitrarily pattern a substrate with a hydrophobic surface modifier. Fluorescent polymers were spin-cast onto the prepatterned substrate to form a homogeneous thin film. Upon exposure to a binary vapor of good- and nonsolvents with an appropriate mixing ratio (cosolvent vapor annealing, coSVA), the polymer film spontaneously transforms into an array of microdisks. This technique does not involve any developing or molding processes, which drastically reduce the difficulty and complexity of the patterning procedure in comparison to the typical lithography process. Moreover, the fluorescence of the microdisks can be switched by doping photochromic fluorescent diarylethenes, which alter the fluorescence of the host polymer via a photoinduced energy transfer. The rich functionalities that are achievable with the coSVA method are highly valuable for constructing micrometer-scale optical memories, displays and sensing devices.

## 2. Materials and Methods 

### 2.1. Materials and Settings

**Figure 1:**
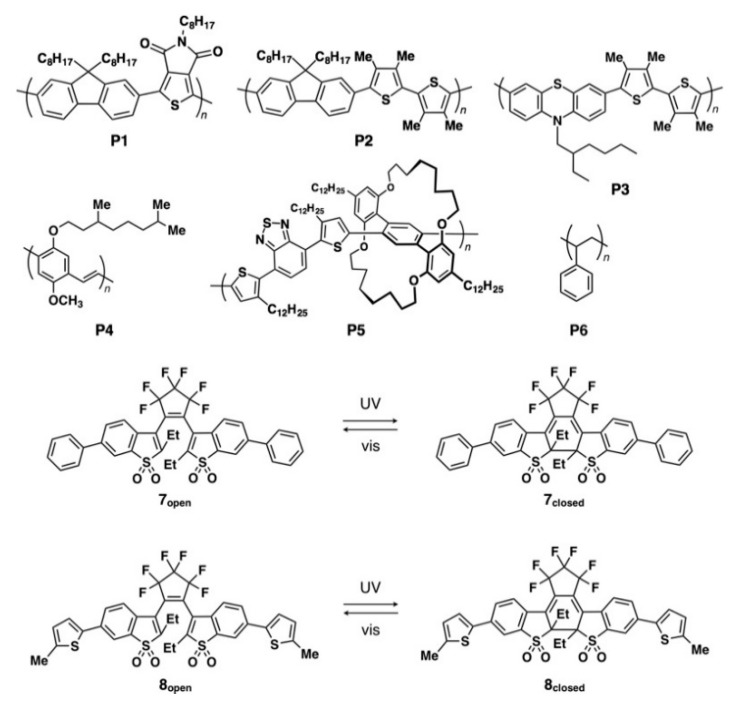
Molecular structures of π-conjugated polymers **P1**–**P5**, nonconjugated polystyrene **P6** and photochromic diarylethenes **7** and **8**.

π-Conjugated polymers **P1** (poly[(9,9-dioctylfluorene-2,7-diyl)-*alt*-(5-octylthieno[3,4-*c*]pyrrole-4,6-dione-1,3-diyl)], number-averaged molecular weight (*M*_n_) = 43,000 g mol^−1^, polydispersity index (PDI) = 2.36), **P2** (poly[(9,9-dioctylfuorenyl-2,7-diyl)-*alt*-(3,3′,4,4′-tetramethylbithiophene-2,5′-diyl)], *M*_n_ = 31,800 g mol^−1^, PDI = 2.46), **P3** (poly[(N-(2-ethylhexyl)phenothiazine-3,7-diyl)-*alt*-(3,3′,4,4′-tetramethylbithiophene-2,5′-diyl)], *M*_n_ = 21,000 g mol^−1^, PDI = 2.82), **P5** (an alternating copolymer containing a phenylene moiety covered with 1,10,17,26-tetraoxa[10.10]metacyclophane as one part and 3-*n*-dodecylthiophene-*alt*-benzothiadiazole-3′-*n*-dodecylthiophene as the counterpart, *M*_n_ = 8600 g mol^−1^, *M*_w_/*M*_n_ = 1.2), and photochromic diarylethenes **7** and **8** were synthesized according to the reported procedures (Figure 1) [9,10,11,12,13,14,15,16,17]. π-Conjugated polymer **P4** (poly[2-methoxy-5-(3′,7′-dimethyloctyloxy)-1,4-phenylenevinylene]) and nonconjugated polystyrene **P6** were purchased from Aldrich Co. Ltd. (Figure 1). Unless otherwise noted, all solvents and reagents were used as purchased. Steady-state photoluminescence (PL) spectra were measured on a JASCO FP-6200 spectrofluorometer. Optical and fluorescent microscopic observations were carried out using an Olympus model BX53 Upright Microscope. Quartz and SiO_2_ (200 nm)-covered silicon (Si) were used as a substrate. Atomic force microscopy (AFM) measurements were conducted on SII-Nanotechnology model S-image scanning probe microscopy.

### 2.2. Cosolvent Vapor Annealing (coSVA)

**Figure 2:**
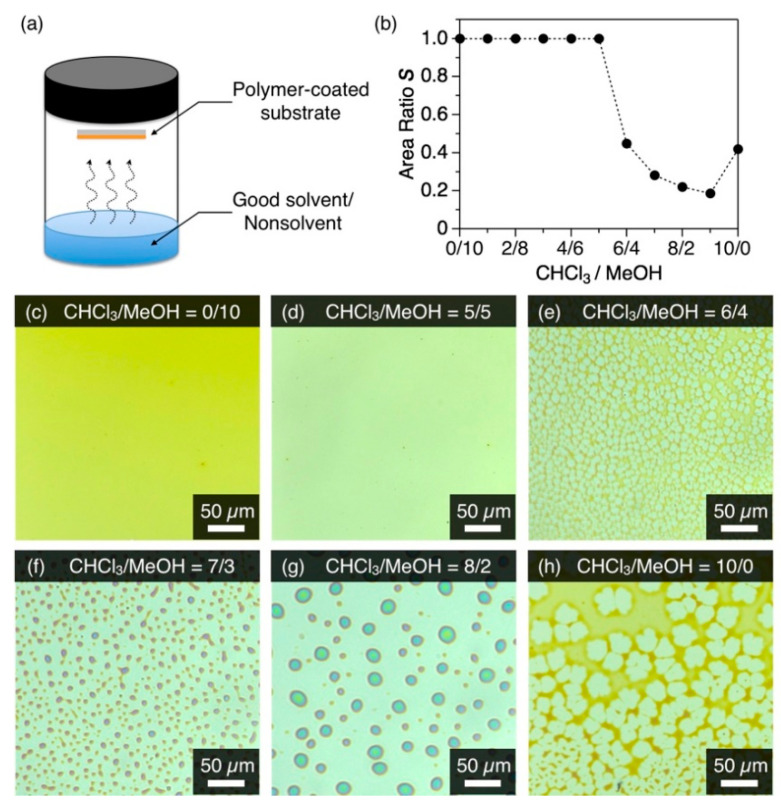
(**a**) Schematic representation of cosolvent vapor annealing (coSVA) method. (**b**) Plot of the area ratio *S* of **P1** versus CHCl_3_/MeOH (*v*/*v*) utilized for coSVA. (**c**–**h**) Optical microscope images of spin-cast films of **P1** after coSVA for 4 h with CHCl_3_/MeOH of (**c**) 0/10, (**d**) 5/5, (**e**) 6/4, (**f**) 7/3, (**g**) 8/2, and (**h**) 10/0.

Typically, 1 mg of conjugated polymer was dissolved in CHCl_3_ (1 mL), and the solution (20 µL) was spin-cast on a quartz or Si substrate (2000 rpm, 40 s). The thickness of the polymer film was 3–10 nm. A 50-mL vial containing 0.6 mL of binary solution of CHCl_3_ and MeOH was sealed with a cap, where a polymer-coated substrate was adhered at the backside of the cap. Then, the vial stood at 30 °C for 1 to 18 h to carry out the cosolvent vapor annealing (coSVA, Figure 2a).

### 2.3. Hydrophobic/Hydrophilic Micropatterning on a Substrate

A quartz or Si substrate was immersed into a CHCl_3_ solution (5 mL) containing hexamethyldisilazane (HMDS, 10 µL). After 12 h, the substrate was fully covered with a monolayer of methyl group that enhanced the hydrophobicity of the surface. The hydrophobic/hydrophilic cross micropattern was fabricated by irradiating parallel vacuum ultraviolet (PVUV) light (*λ* = 150–200 nm, 15 Hz, 130 s, pulse duration: 10 ns) through a photomask with chromium cross-pattern (width of the boxes and lines: 3 and 2 µm, respectively). The intense PVUV light selectively removes the methyl group on the substrate to form a hydrophilic box array pattern on the surface (Figure S1) [18].

## 3. Results and Discussions

### 3.1. Fabrication of Microstructures by coSVA Method

We previously reported that conjugated polymers **P1**–**P5** (Figure 1) self-assemble into microspherical particles upon sluggish precipitation from solution, which is driven by the diffusion of nonsolvent into the polymer solution [19,20,21,22]. However, it is difficult to arrange the resultant microspheres into a regular array on a substrate. To overcome this problem, we attempt the direct transformation of the morphology of the polymers from a thin film to minute disks on a substrate surface by the solvent vapor annealing (SVA) method [23].

A spin-cast film of **P1** on a bare quartz substrate was exposed to a binary mixture of CHCl_3_ and MeOH vapor for the coSVA method. When the composition of the vapor, CHCl_3_/MeOH (*v*/*v*), was less than 6/4, the thin film hardly displayed a morphological change even after a 4 h-exposure to the vapor (Figure 2c,d). In contrast, the thin film transformed into a reticular or dotted pattern when CHCl_3_/MeOH was larger than 6/4 (Figure 2e–h). The morphological transformation of the film upon coSVA was quantitatively assessed in terms of the area ratio *S*, which was given by the coverage area of the film **P1** divided by the whole area observed. The plot of *S* against the CHCl_3_/MeOH ratio showed a clear threshold at CHCl_3_/MeOH = 6/4 and immediately hit a plateau thereafter with a coverage ratio of 0.2 (Figure 2b and Figure S2). When only CHCl_3_ vapor was exposed, the *S* value increased slightly to 0.4. Considering that CHCl_3_ is a good solvent for **P1**, the transformation of the film at a higher CHCl_3_ content is triggered by the partial dewetting of the film with CHCl_3_ and subsequent fluidization. MeOH, a nonsolvent for **P1**, does not fluidize the film but plausibly accelerates the transformation of the film into a separated microdisk morphology.

**Figure 3:**
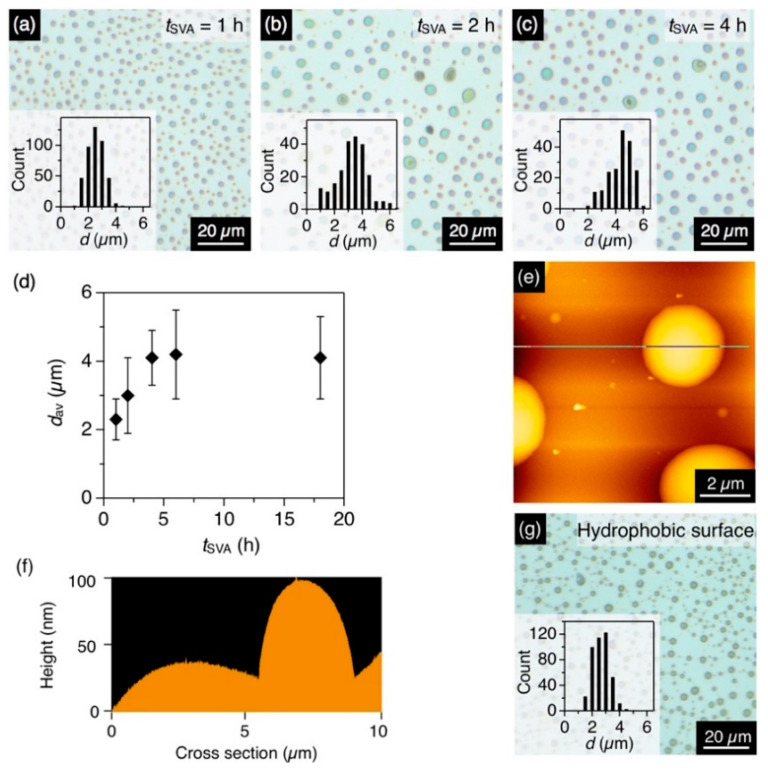
(**a**–**c**) Optical micrographs of the spin-cast films of **P1** with a *t*_SVA_ of (**a**) 1 h, (**b**) 2 h and (**c**) 4 h. The CHCl_3_/MeOH ratio is 1/0.5. Insets show histograms of *d* of the resultant microdisks. (**d**) Plot of *d*_av_ of the microdisk of **P1** versus *t*_SVA_. (**e**,**f**): (**e**) AFM image and (**f**) its cross-section profile of the microdisk of **P1**. (**g**) Optical micrographs of the spin-cast films of **P1** on an HMDS-treated hydrophobic quartz substrate with a *t*_SVA_ of 4 h. The CHCl_3_/MeOH ratio is 1/0.5. The inset shows histograms of *d* of the resultant microdisks.

The time-dependent observation of the structural transformation was conducted with a binary vapor (CHCl_3_/MeOH = 1/0.5, *v*/*v*). After 1 h of coSVA, microdisks formed with an average diameter (*d*_av_) of 2.3 µm and a standard deviation (*σ*) of 0.6 µm (Figure 3a). Upon prolonging the annealing time (*t*_SVA_), *d*_av_ increased and hit a plateau of 4.1 µm (*σ* = 0.8 µm) in 4 h (Figure 3b–d). The average height of the microdisks was ~100 nm according to the height profiles of the AFM images (Figure 3e,f).

A hydrophobic quartz substrate, treated with HMDS, also afforded microdisks upon coSVA (CHCl_3_/MeOH = 1/0.5 *v*/*v*), while the *d*_av_ of the resultant microdisks was 2.4 µm, which was smaller than that observed on a bare quartz substrate (4.0 µm, Figure 3g). This is because of the lesser affinity of the hydrophobic surface with **P1**. **P1** tend to aggregate with each other tightly to form smaller microdisks on the hydrophobic surface.

### 3.2. Fabrication of Microstructures by coSVA Method

**Figure 4:**
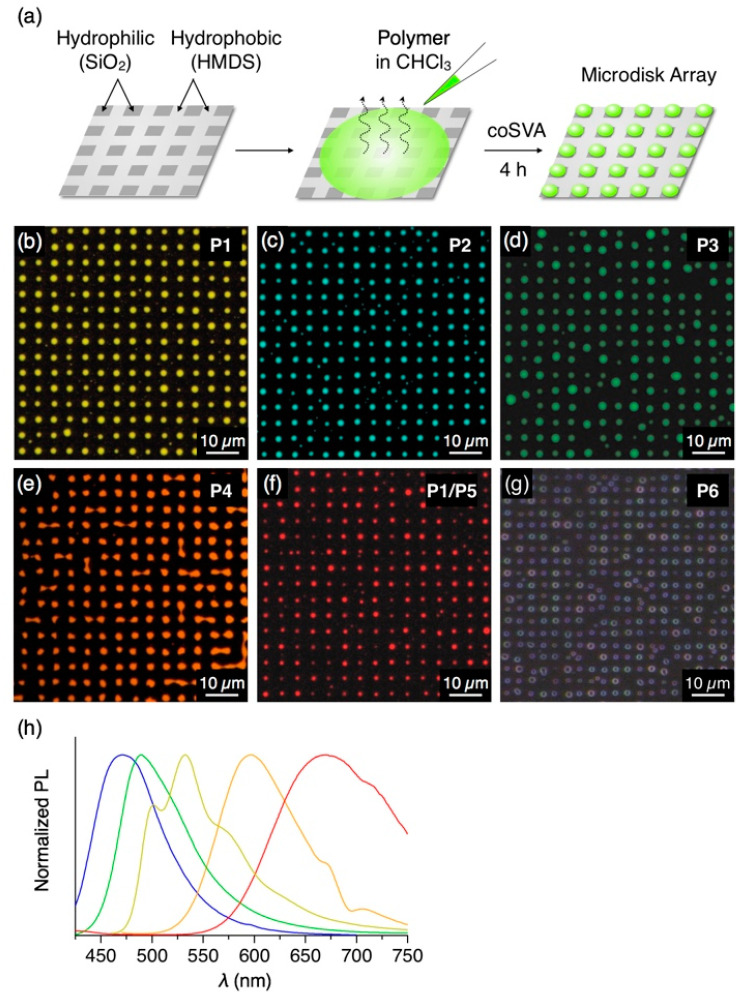
(**a**) Schematic representation of the preparation procedure of the microdisk array of **P1**–**P6** by coSVA on a hydrophobic/hydrophilic patterned substrate. (**b**–**f**) Fluorescent micrographs (*λ*_ex_ = 400–440 nm) of microdisk arrays of (**b**) **P1**, (**c**) **P2**, (**d**) **P3**, (**e**) **P4**, (**f**) **P1**/**P5** (7/3 *w*/*w*) and (**g**) **P6** prepared by coSVA of the corresponding spin-cast films with *t*_SVA_ = 4 h. (**h**) PL spectra (*λ*_ex_ = 400 nm) of thin films of **P1** (yellow), **P2** (blue), **P3** (green), **P4** (orange) and **P1**/**P5** (red) prepared by drop-cast from CHCl_3_ solutions of polymers on a quartz substrate.

Patterned surfaces of quartz and Si substrates were prepared by methylation of the surface and subsequent selected-area demethylation by PVUV irradiation through a patterned photomask. The hydrophobically modified substrate was fabricated as described in Section 2.3. PVUV light was irradiated to the substrate through a cross-patterned photomask. The methyl group on the substrate was photochemically removed upon exposure to PVUV light to form hydrophilic box patterns (Figure 4a and Figure S1). The patterned substrate was then coated with polymer **P1**, for instance, and subjected to coSVA to form a regular array (Figure 4b). 

In an analogous manner to **P1**, π-conjugated polymers **P2**–**P4**, featuring blue, green and orange fluorescence, respectively (Figure 4h), were treated with the coSVA method, successfully resulting in the formation of microdisk arrays (CHCl_3_/MeOH = 1/0.5, annealing time: 4 h, Figure 4c–e). In contrast, **P5** with red PL color hardly yields well-ordered microdisk arrays, despite the fact that **P5** forms microspheres by a vapor diffusion self-assembly process in solution [21]. Meanwhile, **P5** has a high miscibility with other π-conjugated polymers [12] and features an absorption band in 400–550 nm overlapping with the PL band of **P1** (Figure S3), which is advantageous for using **P5** as a dopant of **P1** to give red PL through energy transfer from **P1** to **P5**. With this concept in mind, we conducted coSVA with a spin-cast film of a mixture of **P1** and **P5** (7/3 *w*/*w*) and found the formation of the analogous microdisk arrays (Figure 4f). By doping **P5** with a weight ratio of **P5** of more than 20% (Figure S4), the fluorescent color of the microdisks turns to red via photoinduced energy transfer from **P1** to **P5**. Nonconjugated polystyrene **P6** also forms microdisk arrays via the coSVA method (Figure 4g).

### 3.3. Fluorescence Switching and Color Change

The turn-on fluorescence property and its color switching are of practical importance, especially for the development of optical displays and memories. To this end, we incorporated photoswitchable diarylethenes **7** and **8** as fluorescent dopants into the polymer media. Closed-form isomers of **7** and **8** (**7**_closed_ and **8**_closed_) are emissive with yellow and red colors, respectively, while their open-form isomers (**7**_open_ and **8**_open_) are nonemissive under excitation with 400–440-nm light [13,14,15,16,17].

**Figure 5:**
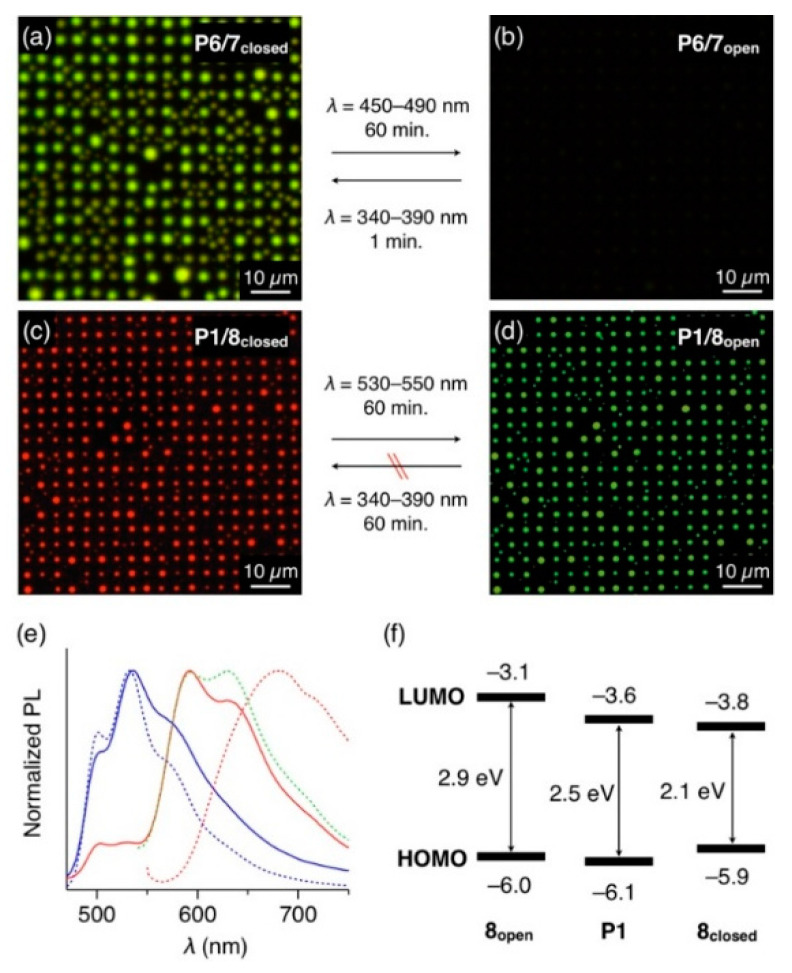
(**a**,**b**) Fluorescent micrographs (*λ*_ex_ = 400–440 nm) of microdisk arrays of **7_closed_**-doped (10 wt.%) **P6** (**a**) before and (**b**) after photoirradiation at 450–490 nm. (**c**,**d**) Fluorescent micrographs (*λ*_ex_ = 400–440 nm) of microdisk arrays of **8_closed_**-doped (10 wt.%) **P1** (**c**) before and (**d**) after photoirradiation at 530–550 nm. (**e**) PL spectra (*λ*_ex_ = 400 nm) of thin films of **P1** doped with 10 wt.% of **8**_open_ (blue, solid line) and **8**_closed_ (red, solid line), prepared by drop-cast of CHCl_3_ solutions of **P1** and **8** on a quartz substrate. The dashed lines show PL spectra of thin films of **P1** (blue) and **8**_closed_ (red). The green dashed line shows the PL spectrum of a CHCl_3_ solution of **8**_closed_. (**f**) HOMO and LUMO energy levels of **P1**, **8**_open_, **8**_closed_, obtained from a cyclic voltammogram.

Luminescence on/off switching of the polymer microdisk arrays was achieved from arrays of **P6** doped with **7**. A mixture of **P6** and diarylethene **7**_closed_ (10 wt.%) dissolved in CHCl_3_ was spin-cast on a patterned substrate. The resultant film was then subjected to coSVA (CHCl_3_/MeOH = 1/0.5) to form a microdisk array with yellowish-green PL (Figure 5a). Upon photoirradiation (*λ* = 450–490 nm) for 60 min, **7**_closed_ photo-isomerized into **7**_open_, and the microdisks turned nonemissive (Figure 5b). Conversely, upon irradiation of UV light (*λ* = 340–390 nm), fluorescence was recovered within 1 min by the backward photo-isomerization. 

Since **8**_closed_ acts as an energy acceptor for **P1**, the PL color of **P1**/**8** mixed film is switchable by the photoisomerization of **8**. In fact, the **8**_closed_-doped **P1** microdisk array displays red PL upon photoexcitation of **P1** at *λ*_ex_ = 400–440 nm (Figure 5c). The PL spectrum of the microdisks matches well with those of the solution of **8**_closed_, not with the aggregate state of **8**_closed_, indicating that **8**_closed_ is well-dispersed in the **P1** medium and emits luminescence via energy transfer from **P1** (Figure 5e). Upon photoirradiation at 530–550 nm, **8**_closed_ isomerized into nonemissive **8**_open_. Accordingly, the PL color of the array changed from red to yellowish green, which was attributed to the PL of **P1** (Figure 5d). However, the reverse photoisomerization reaction (from **8**_open_ to **8**_closed_) barely occurred in the **P1** matrix, even with a 60-min exposure to UV light. This is possibly because energy transfer from **8**_open_ to **P1** occurs prior to the photoisomerization of **8**_open_ to **8**_closed_, considering the HOMO and LUMO energy levels of **P1** and **8_open_** (Figure 5f), determined by their cyclic voltammetry (Figure S5) and photoabsorption spectra (Figure S6).

## 4. Conclusions

The newly developed cosolvent vapor annealing (coSVA) method is effective for the fabrication of regularly deposited microdisks. The morphology of the resultant microaggregates is tunable by changing the mixing ratio of good- and nonsolvents. The arrangement of the aggregates is arbitrarily controlled by the hydrophobic/hydrophilic micropatterned substrate over a whole substrate surface as large as a square centimeter scale. The turn-on/off of PL of the microdisks is switchable by doping photochromic molecules. Furthermore, the fluorescence color of the π-conjugated polymer microarray can be switched upon photoisomerization. The self-assembled fluorescent microdisk arrays with several micrometer periodic patterns will be applicable to future optical memories and displays, as well as tools for chemical and biological sensing.

## 5. Patents

Japanese patent applications 2019-021022 and 2018-016723, and Japanese patent B6420092.

## Data Availability

The data presented in this study are available on request from the corresponding author.

## Supplementary Materials

The following is available online at https://www.mdpi.com/2073-4360/13/2/269/s1, Figure S1: fabrication of patterned substrates, Figure S2: microscopic images, Figure S3: electronic absorption and PL spectra, Figure S4: fluorescent microscopy images, Figure S5: cyclic voltammograms, Figure S6: electronic absorption spectra.
