# Supplementary material for: Fluorescence Switchable Conjugated Polymer Microdisk Arrays by Cosolvent Vapor Annealing
Source: Polymers (Basel). 2021 Jan 15;13(2):269. doi: 10.3390/polym13020269 (PMC7829903; doi:10.3390/polym13020269)
Supplement: Supplementary file 1 [file polymers-13-00269-s001.pdf]

Supporting Information

Fluorescence switchable conjugated polymer microdisk arrays by cosolvent vapor annealing

Hiroshi Yamagishi , Tokiya Matsui, Yusuke Kitayama , Yusuke Aikyo , Liang Tong , Junpei Kuwabara, Takaki Kanbara , Masakazu Morimoto, Masahiro Irie , and Yohei Yamamoto *

Department of Materials Science, Faculty of Pure and Applied Sciences, University of Tsukuba, 1-1-1 Tennodai, Tsukuba, Ibaraki 305-8573, Japan.

Tsukuba Research Center for Energy Materials Science (TREMS), Faculty of Pure and Applied Sciences, University of Tsukuba, 1-1-1 Tennodai, Tsukuba, Ibaraki 305-8573, Japan.

Department of Chemistry, Rikkyo University, 3-34-1 Nishi-Ikebukuro, Toshima, Tokyo 171-8501, Japan.

*Correspondence and requests for materials should be addressed to Yohei Yamamoto (yamamoto@ims.tsukuba.ac.jp).

1. Materials

Commercial reagents were purchased from Sigma-Aldrich, TCI, and Wako Pure Chemical Industries, Ltd. All the chemicals are used as received unless otherwise mentioned.

2. General

Light irradiation was conducted with a Hamamatsu model LC-L1V5 UV-LED spot light source. Electronic photoabsorption spectra were measured on a JASCO model V-570 spectrophotometer. Steady-state photoluminescence (PL) spectra were measured on a JASCO model FP-6200 spectrofluorometer with band width of 5 nm. Optical and fluorescent microscopic observations were carried out using an Olympus model BX53 Upright Microscope. SEM microscopy was performed on a Hitachi model S-3700N SEM operating at 30 kV. Atomic force microscopy (AFM) measurements were conducted on a SII-Nanotechnology model S-image scanning probe microscopy. CV measurements were carried out on ALS model 600C electrochemical analyzer with Pt as working and counter electrodes and Ag/Ag^{+} on Pt as a reference electrode. Hydrophilic/hydrophobic micropatterns were prepared with Ushio Inc. model SUS740 parallel vacuum/UV light (150–200 nm wavelength).

3. Fabrication of patterned substrate

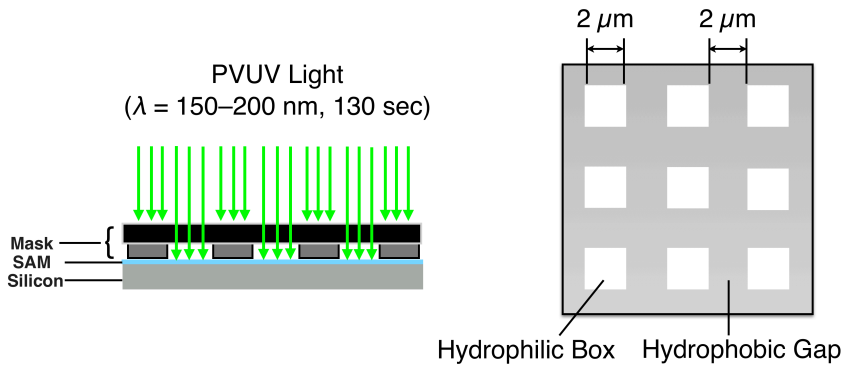

Figure S1. Fabrication procedure for the patterned substrate and a schematic representation of the resultant substrate with hydrophilic boxes and hydrophobic gaps.

4. Microscopic images of P1 after coSVA

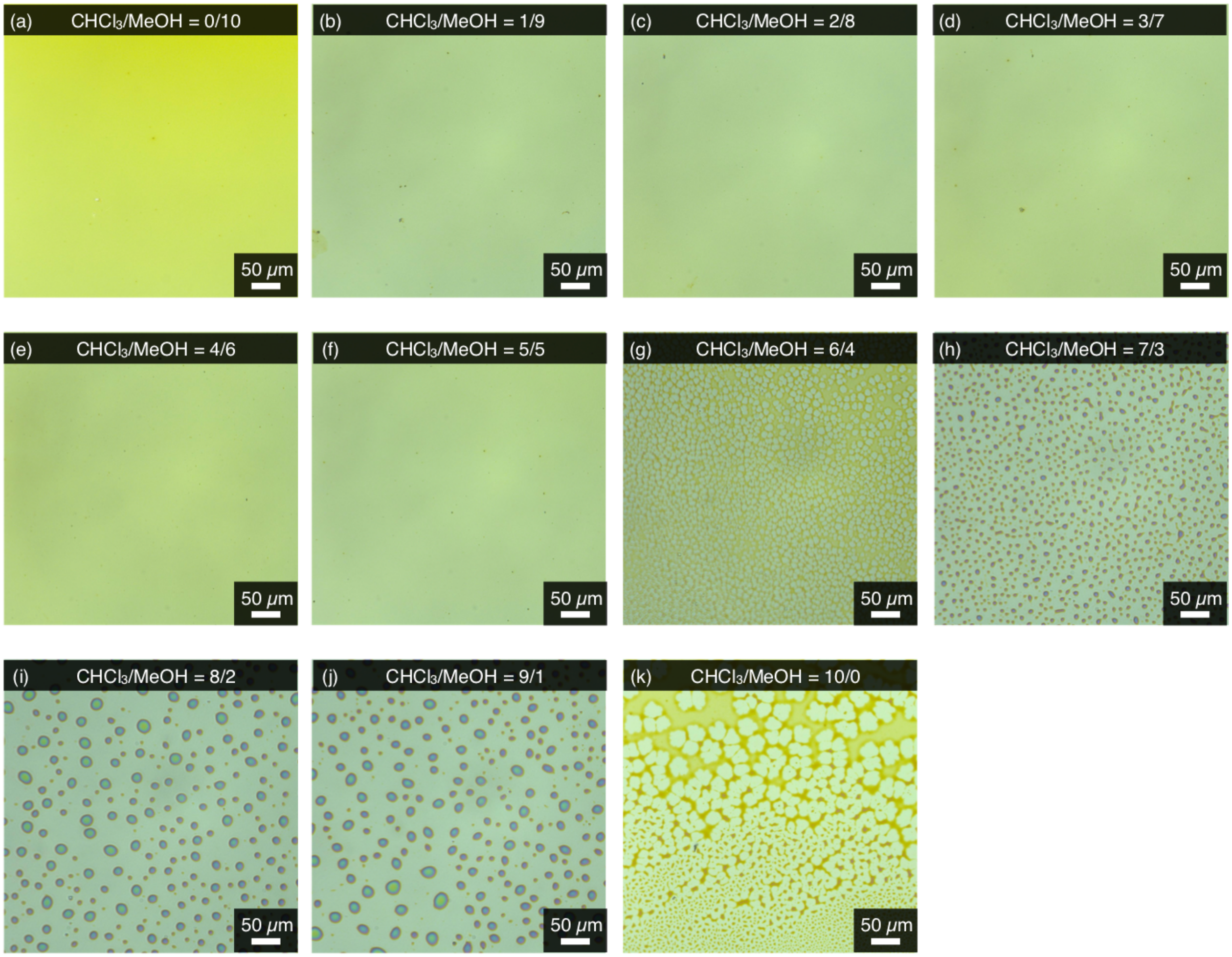

Figure S2. Optical microscope images of spin-cast films of **P1** after coSVA for 4h with $CHCl_3$/MeOH of 0/10 (a), 1/9 (b), 2/8 (c), 3/7 (d), 4/6 (e), 5/5 (f), 6/4 (g), 7/3 (h), 8/2 (i), 9/1 (j), and 10/0 (k).

5. Electronic absorption spectrum of P1 and photoluminescence spectrum of P5

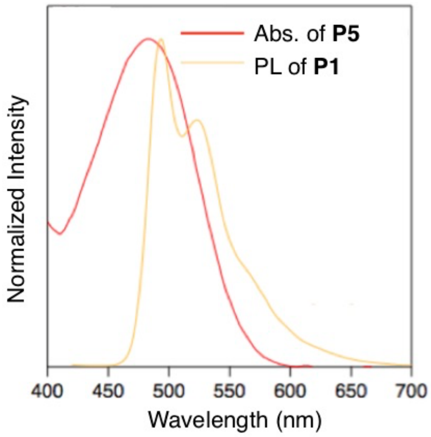

Figure S3. An electronic absorption spectrum of **P5** in $CHCl_3$ (red curve) and a PL spectrum of **P1** in $CHCl_3$ (yellow curve, λ_{ex} = 400 nm).

6. Fluorescent microscopic images

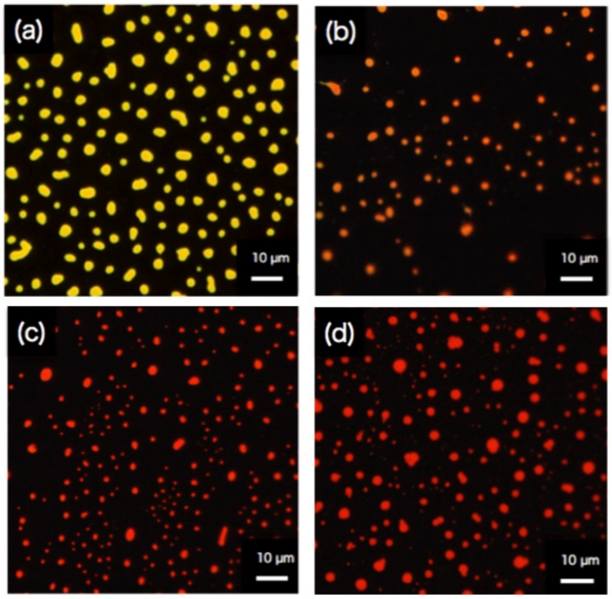

Figure S4. Fluorescent microscopic images of microdisks of **P1** and **P5** mixed together with weight ratio of 9/1 (a), 8/2 (b), 7/3 (c), and 5/5 (d).

7. Cyclic voltammetry of $\mathbf{8_{close}}$ and $\mathbf{8_{open}}$

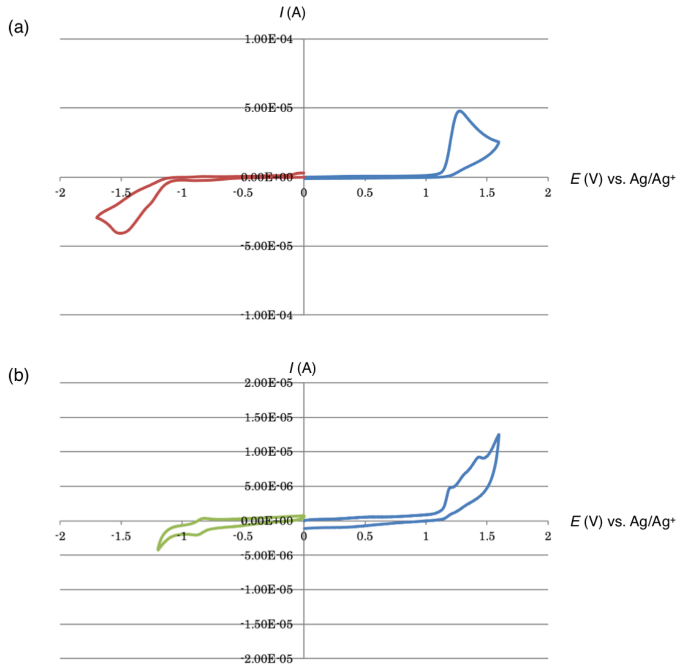

Figure S5. Cyclic voltammograms of $\mathbf{8_{open}}$ (a) and $\mathbf{8_{close}}$ (b) measured in MeCN (2 mM) containing NBu_4ClO_4 (0.1 M) as an electrolyte, featuring redox half potentials E_{red} of –1.590 V and E_{ox} of +1.194 V for $\mathbf{8_{open}}$ and E_{red} of –0.973 V and E_{ox} of +1.122 V for $\mathbf{8_{close}}$ (vs. Fc/Fc^+).

8. Electronic absorption spectra of $\mathbf{8_{close}}$ and $\mathbf{8_{open}}$

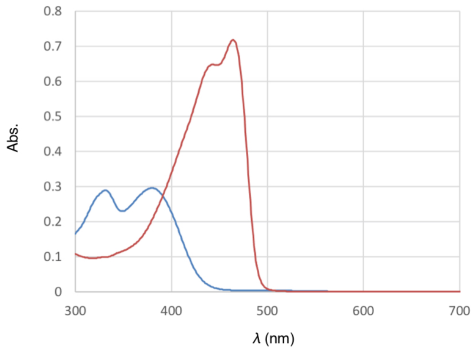

Figure S6. Electronic absorption spectra of $CHCl_3$ solution of $\mathbf{8_{open}}$ (blue) and **P1** (red).
